# Recreational cannabis use in hemodialysis patients: prevalence, chronic pain profile and impact on quality of life

**DOI:** 10.1186/s42238-026-00471-3

**Published:** 2026-08-03

**Authors:** Fatima Zahra Bouchachi, Nadia AL Wachami, Younes Iderdar, Maryem Arraji, Karima Boumendil, Chaimaa El Moubchiri, Hasna Zaeria, Abdeljalil El Got, Zineb Serhier, Mohamed Chahboune

**Affiliations:** 1 Hassan First University of Settat, Higher Institute of Health Sciences, Laboratory of Health Sciences and Technologies, P.O. Box : 539, Settat, 26000 Morocco; 2 Hassan II University of Casablanca, Faculty of Medicine and Pharmacy, Clinical Neuroscience and Mental Health Laboratory, P.O. Box: 9154, Tarik Ibnou Ziad Street, Casablanca, 20250 Morocco

**Keywords:** Chronic pain, Cannabis, Hemodialysis, Quality of life, Smoking

## Abstract

**Background:**

Morocco is one of the world's leading suppliers of cannabis, primarily cultivated in its northern region. Smoked cannabis has been reported in the literature to relieve suffering. Pain is a prevalent symptom in hemodialysis patients, leading to decreased quality of life. Therefore, this study aims to determine the prevalence of smoking cannabis in male patients undergoing hemodialysis and to explore the sociodemographic, biological and chronic pain characteristics, and quality of life between users of recreational cannabis and non-users.

**Methods:**

A total of 186 male patients on hemodialysis were recruited from four dialysis centers in the northern Morocco region. The Numeric Rating scale was used to assess pain severity and the Short-Form 36 (SF-36) questionnaire was adopted to evaluate the quality of life. The Brief Pain Inventory was used to locate the painful sites. Data collection utilized medical files and a structured questionnaire including sociodemographic, clinical and dialysis-related variables.

**Results:**

The prevalence of cannabis use was 18.2%. Patients in rural areas and current tobacco smokers use more cannabis. Compared to the non-smokers' group, the smokers' group had less chronic pain (p = 0.002) and lower pain severity (p = 0.006). For the quality of life, significantly higher mean scores in general health, bodily pain and vitality domains (p = 0.02), (p < 0.001) and (p = 0.009) respectively, and increased physical component summary (p = 0.003). Furthermore, cannabis smokers were more Vitamin D deficient (p = 0.009) and had a higher serum concentration of hemoglobin (p = 0.005) compared to non-smokers.

**Conclusions:**

Cannabis smokers were predominantly from rural areas, had a history of tobacco use and were less likely to suffer from chronic pain than non-smokers. The smoker's group had low pain severity and better quality of life compared to non-smokers. However, more research is needed to investigate the relationship of frequency, quantity and quality of cannabis on efficiency and reduction of chronic pain in patients treated by hemodialysis.

## Background

Cannabis is a flowering plant comprising three primary species (*indica*, *sativa* and *ruderalis*), which are distinguished by their genetic and chemical capacity to produce varying concentrations of phytocannabinoids (Hillig and Mahlberg 2004). To date, nearly 150 different cannabinoids have been identified, with the two most prominent being Δ−9-tetrahydrocannabinol (THC) and cannabidiol (CBD) (Citti et al. 2019). THC is a psychoactive compound responsible for the behavioral effects (Citti et al. 2019); however, CBD is the second most plentiful cannabinoid and the main non-euphoric agent (Mlost et al. 2020). The two components have antinociceptive properties (Schott 2019; Costa et al. 2014).

Cannabis is used as a medical drug or dried plant consumed as a psychoactive substance. Its therapeutic benefits are demonstrated in various conditions (Bilbao and Spanagel 2022) and symptom management (Santos et al. 2021). Cannabis is used in chronic kidney disease (CKD) and patients treated by hemodialysis to control pain and to improve sleep quality (Collister et al. 2023). Some hemodialysis patients reported its use for anxiety, mood and appetite (Ho et al. 2024). In addition, preliminary evidence appears to be encouraging its use for restless legs syndrome and uremic pruritus in this population (Samaha et al. 2020).

Cannabis is cultivated in numerous countries; Morocco stands out as one of the world's leading suppliers of cannabis (World Drug Report 2022). Its cultivation in Morocco dates back to the late 16th century, initially confined to a small region before expanding to the northern part of the country. In 2021, a law on the legal use of cannabis was adopted to authorize the cultivation of cannabis for medical and industrial purposes (Moroccan Government 2021). Today, 86% of cannabis cultivation is concentrated in three provinces: Chefchaouen (50%), Taounate (19%) and Al Hoceima (17%) (2003). Moroccan cannabis is of Sativa type, characterized by a high concentration of THC, rising from 0.6–16.8% in 2005 to 0.5–25% in 2014 (Stambouli et al. 2016), with a THC/CBD ratio of 2:1 (2016). Cannabis can be consumed in two derivative forms. kif (marijuana or cannabis herb) refers to dried and crushed leaves of the cannabis plant, which can be smoked alone or combined with tobacco using a Moroccan pipe (sebsi). Hashish is obtained by extracting resin from the dried flowers of the cannabis plant (Abdelaziz et al. 2016) that can be smoked in conjunction with tobacco. Kif is the most frequently used in northern Morocco due to its availability and low cost.

Smoked cannabis has been reported in the literature to relieve suffering (Wong et al. 2020). Chronic pain is a prevalent symptom in end-stage kidney disease (ESKD) patients undergoing hemodialysis, leading to decreased quality of life (Ishtawi et al. 2023). Hemodialysis patients with chronic pain have been found to have anxiety, depression, alteration in hemodialysis adherence and the use of medical resources (Weisbord et al. 2014). In addition**,** the pain was associated with the incidence of morbidity and mortality in this population (Kliuk-Ben Bassat et al. 2019). Despite the advent of pharmacological and non-pharmacological treatments, it is difficult to obtain sufficient pain relief for these patients (Kliuk-Ben Bassat et al. 2019).

Some evidence investigated the use of cannabis in CKD. A recent review provides an overview of cannabis and cannabinoids with relevance to the practice of nephrology (Rein 2020). This research shows that cannabis may have benefits for treating pain in CKD and ESKD. In addition, cannabis does not seem to affect kidney function in healthy people; however, renal function should be closely monitored in those with CKD (Rein 2020). Moreover, cannabis usage is becoming more widespread, regardless of one's political, social or personal beliefs (Rein and Wyatt 2018). Consequently, nephrologists should be aware of the possible effects on their patient group (Rein and Wyatt 2018). Nevertheless, a few studies have examined cannabis use for pain management in hemodialysis patients. Kliuk-Ben Bassat’s randomized research evaluated the safety of sublingual cannabis oil for pain management in this population (Kliuk-Ben Bassat et al. 2023); however, the relationship between cannabis use and chronic pain has not been assessed. Ho’s study examined the characteristics and perspectives of cannabis use among patients undergoing hemodialysis (Ho et al. 2024); Nonetheless, the research included both medical and recreational cannabis, involved a limited sample of only 52 participants and did not establish the relationship with pain management. Furthermore, while Ho’s review focused on CKD, the results discussed the effectiveness of cannabis for chronic pain in non-renal diseases (Ho et al. 2019), due to insufficient clinical research, especially involving CKD patients. The use of cannabis in the management of pain in hemodialysis patients has been evaluated (Ho et al. 2024; Kliuk-Ben Bassat et al. 2023); however, the inhaled form must be specifically evaluated, as it is known for its dose-dependent pharmacokinetics in reducing neuropathic pain intensity (Almog et al. 2020). Moreover, in Morocco, the prevalence of smoking cannabis, pain characteristics and quality of life related to its use in these subjects are unknown. The objective of this research was to determine the prevalence of smoking cannabis in male patients undergoing hemodialysis and to explore sociodemographic, biological and chronic pain characteristics and quality of life between users of recreational marihuana and non-users.

## Methods

### Study design and setting

This study employed a cross-sectional design to evaluate the prevalence of cannabis use among male hemodialysis patients and to examine the differences in chronic pain characteristics and quality of life between recreational cannabis users and non-users within this population. The research was conducted in four hemodialysis centers in northern Morocco, providing two hemodialysis sessions per week, each of which does not exceed five hours.

### Population and sample size

Convenience sampling was employed to recruit male hemodialysis patients from public hemodialysis centers. The sample size was calculated using the Raosoft formula, with a 5% margin of error, a 95% confidence interval and a response distribution of 50%. The minimum required sample size was determined to be 169 participants. A total of 190 patients met the eligibility criteria and were included in the study.

### Inclusion and non-inclusion criteria

Participants who met the following criteria were included in the study: male patients undergoing chronic hemodialysis, aged 18 years or older, with a duration of hemodialysis treatment of at least 6 months at the time of the study and who provided informed consent to participate. Subjects were excluded if they had a cancer diagnosis, presented with mental or hearing disorders, used other forms of cannabis (non-smoked), or were female. Cancer patients were excluded from the study due to the overlap and difference in pain physiology between hemodialysis and cancer patients. Additionally, those with mental or hearing impairments were excluded to prevent communication issues and misunderstandings. Women were excluded from the study due to a combination of sociocultural and epidemiological factors, without any intentional selection bias. From a sociocultural perspective, within the Moroccan context, cannabis use is more socially accepted among men than among women. This disparity in social norms may lead women to underreport such behavior due to stigma, fear of judgment, or potential family-related repercussions, resulting in non-disclosure or reluctance to participate in the study. From an epidemiological standpoint, available data consistently indicate a significantly higher prevalence of cannabis use among men in the general population.

### Data collection instrument

Data were collected using a structured questionnaire divided into three sections. The information was collected through face-to-face interactions between the participants and the investigator. The first section focused on sociodemographic characteristics, including age, residence, educational level, marital status, occupation, monthly income and lifestyle. Tobacco smoking status was categorized as follows: Daily smoker (who smokes a tobacco product at least once a day), occasional smoker (a person who smokes a tobacco product irregularly and not on a daily basis, in the last six months), Ex-smoker (any smoker who has stopped smoking at least six months prior to the study) and non-smoker (who has never used tobacco).

Cannabis consumption was defined as follows: a cannabis smoker was any participant who had used cannabis within the last three months, while a non-cannabis smoker was defined as someone who had never used cannabis or had stopped using it at least three months prior to the study. Frequency has been further assessed: once or twice in the last 3 months, monthly (1–3 times per month), weekly (1–4 times per week), daily or almost daily (5–7 days per week).

The second section covered clinical features, including Body Mass Index (BMI) and the etiology of ESKD. Dialysis-related variables were also recorded, such as interdialytic weight gain (in kilograms, representing weight gain between two hemodialysis sessions), dialysis duration (in hours), dialysis vintage (in years), frequency of sessions per week, and type of vascular access: arteriovenous fistula (AVF) proximal, AVF distal, or tunneled catheter. The third section included biological analyses, such as levels of 25-hydroxyvitamin D (25(OH)Vit D), C-reactive protein (CRP), hemoglobin (HB) and parathyroid hormone (PTH). Regarding treatment, the protocol for managing anemia in hemodialysis patients focuses primarily on correcting iron deficiency using erythropoiesis-stimulating agents. In addition, for bone management, a rigorous protocol is designed to regulate Vit D and maintain balanced PTH levels, including the prescription of the active vitamin D.

#### Quality of life assessment

Health-related quality of life (HRQoL) was assessed using the Arabic version of the Short-Form 36 (SF-36) questionnaire (Guermazi et al. 2012). The SF-36 is one of the most widely used instruments for measuring the HRQoL in hemodialysis patients, and numerous studies have demonstrated its reliability and validity (Mingardi et al. 1999). This tool consists of 36 items that evaluate the eight health domains of well-being and functioning: physical functioning (PF), role limitations due to physical problems (RP), general health (GH), bodily pain (BP), vitality (VT), role limitations due to emotional problem (RE), mental health (MH) and social functioning (SF) (Ware et al. 1993). Scores for each domain range from 0 to 100, with higher scores indicating better health and/or higher levels of functioning, while lower scores reflect poorer health and/or reduced functioning. The SF-36 includes two summary measures: the Mental Component Summary (MCS) and the Physical Component Summary (PCS). These summary scores are derived from the eight domain scores through a three-step process: standardization of scales (using Z-scores), aggregation of scale scores and transformation of summary scores (Ware et al. 1993).

#### Characteristics of chronic pain

Chronic pain was defined as frequent or persistent interdialytic pain lasting more than three months (Treede et al. 2015). Pain intensity was assessed using the Numeric Rating Scale (NRS), which demonstrated valid psychometric properties and sensitivity in measuring pain intensity in clinical practice (Pagé et al. 2012). NRS allows patients to rate their pain on a scale from 0 to 10. Scores were categorized as follows: 0 for no pain, 1–3 for mild pain, 4–6 for moderate pain and 7–10 for severe pain. The location of pain was identified using the body map from the Brief Pain Inventory (BPI). Multiple sites were assessed, including the upper limbs, lower limbs, neck, shoulders, back, chest, abdomen, hips and knees.

### Ethical consideration

This research was approved by the Committee for Biomedical Research (CERB 28–24) of the Mohammed V Faculty of Medicine and Pharmacy in RABAT. The questionnaire was distributed to the participants, including a written informed consent form explaining the research goal and guaranteeing data privacy and confidentiality.

### Statistical analysis

Data were analyzed using the Statistical Package for the Social Sciences (SPSS) software, version 26. The normality of the data was evaluated using the Kolmogorov–Smirnov test. Categorical variables were described using frequencies and differences between groups were assessed using the Chi-square test or Fisher’s exact test, as appropriate. Quantitative variables were expressed as means ± standard deviation or median (interquartile range), depending on their distribution. Differences between groups for quantitative variables were determined using the Student’s t-test or the Mann–Whitney U test, based on the fulfillment of their respective application conditions. The logistic regression analysis was used to control for confounders. The reliability of the SF-36 questionnaire was evaluated using Cronbach’s alpha. A p-value of < 0.05 was considered statistically significant. Observations with missing data were excluded from the analysis; only complete responses were included.

## Results

### Characteristics of hemodialysis patients

In the study, 190 participants were enrolled. A total of 186 male hemodialysis patients were included in the study. The questionnaire response rate was 97.8% and four patients (2.1%) were excluded because they could not complete the questionnaire for the following reasons: one for headache, two due to cramping and one for arterial hypertension (considered as missing data). Among the non-smokers, five patients use opioids; conversely, among the cannabis smokers, no one consumed opioids. The mean age was 53.4 ± 15.47 years and 75.8% were 65 or less. Most of the participants were from rural areas (61.8%). A significant proportion of the patients were illiterate (65.6%). Furthermore, over three-quarters of the respondents were married (75.8%) and a large majority were unemployed (70.4%). Table 1 displays the sociodemographic and clinical characteristics of cannabis smokers and non-smokers undergoing hemodialysis. Half of the hemodialysis patients had been undergoing treatment for four years or less. Additionally, 84.9% of the subjects had anemia, with 21.5% having hemoglobin levels below or equal to 7.99 g/dl. Regarding vitamin D, 66.1% of the hemodialysis patients showed insufficiency (between 10 and 30 ng/ml) and 13.4% presented with severe deficiency (< 10 ng/ml). The dialysis and biological characteristics of the participants are summarized in Table 2.

**Table 1:** Sociodemographic and clinical characteristics of cannabis smokers and non-smokers undergoing hemodialysis (N = 186)

Variables	N (%)	Cannabis		*P* value
		Non-smoker n (%) n = 152	Smoker n (%) n = 34	
Age		53.81 ± 16.056	51.88 ± 12.627	0.53*
18–49 years	72(38.7)	57(37.5)	15(44.1)	0.35
50–65 years	69(37.1)	55(36.2)	14(41.2)	
> 65 years	45(24.2)	40(26.3)	5(14.7)	
Residence				**0.02**
Urban	71(38.2)	64(42.1)	7(20.6)	
Rural	115(61.8)	88(57.9)	27(79.4)	
Level of studies				0.89
Illiterate	122(65.6)	98(64.5)	24(70.6)	
College	39(21.0)	33(21.7)	6(17.6)	
High school	21(11.3)	17(11.2)	4(11.8)	
University education	4(2.2)	4(2.6)	0(0.0)	
Marital status				0.65
Single	34(18.3)	28(18.4)	6(17.6)	
Married	141(75.8)	114(75.0)	27(79.4)	
Divorced	4(2.2)	3(2.0)	1(2.9)	
Widowed	7(3.8)	7(4.6)	0(0.0)	
Occupation				1
Unemployed	131(70.4)	107(70.4)	24(70.6)	
Employed	52(28.0)	42(27.6)	10(29.4)	
Retired	3(1.6)	3(2.0)	0(0.0)	
Student	0(0.0)	0(0.0)	0(0.0)	
Income per month				0.21
< 297£	177(95.2)	146(96.1)	31(91.2)	
> 297£	9(4.8)	6(3.9)	3(8.8)	
Tobacco smoking status				** < 0.001**
Daily smoker	7(3.8)	4(2.6)	3(8.8)	
Occasional smoker	2(1.1)	2(1.3)	0(0.0)	
Ex-smoker	71(38.2)	48(31.6)	23(67.6)	
Non-smoker	106(57.0)	98(64.5)	8(23.5)	
Etiology of ESKD				0.87
Indeterminate nephropathy	101(54.3)	79(52.0)	22(64.7)	
Vascular Nephropathy	20(10.8)	16(10.5)	4(11.8)	
Diabetic nephropathy	28(15.1)	23(15.1)	5(14.7)	
Chronic interstitial nephropathy	9(4.8)	8(5.3)	1(2.9)	
Glomerular nephropathy	9(4.8)	8(5.3)	1(2.9)	
Polycystic kidney disease	13(7.0)	12(7.9)	1(2.9)	
Lithiasis nephropathy	6(3.2)	6(3.9)	0(0.0)	
BMI (mean ± SD)	23.23 ± 3.99	23.21 ± 3.9	23.35 ± 4.42	0.86*

Chi-square and Fisher tests

Significant result in bold, p < 0.05

*ESKD* End Stage Kidney Disease, *BMI* Body Mass Index, *SD* Standard Deviation

^*^Student t-test

**Table 2:** Dialysis and biological characteristics of cannabis smokers and non-smokers undergoing hemodialysis (N = 186)

Variables	N (%)	Cannabis		*P* value
		Non-smoker n (%) n = 152	Smoker n (%) n = 34	
Dialysis vintage/year				0.24
< 2	50(26.9)	37(24.3)	13(38.2)	
2- 4	43(23.1)	37(24.3)	6(17.6)	
> 4	93(50.0)	78(51.3)	15(44.1)	
Dialysis session duration				0.74
< 4H	17(9.1)	15(9.9)	2(5.9)	
> or equal 4H	169(90.9)	137(90.1)	32(94.1)	
Frequency of sessions				
2 sessions/week	186(100)	152(100)	34(100)	NA
3 sessions/week	0(0)	0(0)	0(0)	
Vascular access				0.79
AVF proximal	29(15.6)	25(16.4)	4(11.8)	
AVF distal	142(76.3)	115(75.7)	27(79.4)	
Tunneled catheter	15(8.1)	12(7.9)	3(8.8)	
Interdialytic weight gain in Kg				1
⩽ 1	6(3.2)	5(3.3)	1(2.9)	
1 < WG ⩽ 3	44(23.7)	36(23.7)	8(23.5)	
> 3	136(73.1)	111(73.0)	25(73.5)	
25(OH)Vit D				
< 10 ng/ml	25(13.44)	19(12.5)	6(17.65)	
10 ⩽Vit D ⩽30 ng/ml	123(66.13)	98(64.47)	25(73.53)	
> 30 ng/ml	38(20.43)	35 (23.03)	3(8.82)	**0.009**
25(OH)Vit D ng/ml (mean ± SD)	23.24 ± 11.96	24.11 ± 12.47	19.34 ± 8.41	
CRP mg/L median (IQR)	3(9.06)	3.1(9.37)	1.05(8.31)	0.14**
HB g/dl				
⩽ 7.99	40(21.5)	35(23.03)	5(14.71)	
8 ⩽HB ⩽11	118(63.4)	98(64.47)	20(58.82)	
> 11	28(15.1)	19(12.5)	9(26.47)	
HB g/dl (mean ± SD)	9.34 ± 1.59	9.19 ± 1.53	10.04 ± 1.67	0.005*
PTH pg/ml (mean ± SD)	439.83 ± 297.61	435.35 ± 292.60	459.85 ± 322.95	0.66*
Vit D intake				
Yes	28(15.05)	22(14.47)	6(17.65)	0.63
No	158(84.95)	130(85.53)	28(82.35)	
Erythropoiesis-stimulating agent use				
Yes	57(30.65)	48(31.58)	9(26.47)	0.56
No	129(69.35)	104(68.42)	25(73.53)	

Chi-square and Fisher tests

Significant result in bold, p < 0.05

*AVF* Arterial Venous Fistula, *Vit D* Vitamin D, *HB* Hemoglobin, *CRP* C-Reactive Protein, *PTH* Parathyroid Hormone, *SD* Standard Deviation, *IQR* Interquartile

*NA* Not applicable

^*^Student t-test

^**^Mann–Whitney test

### Prevalence and characteristics of cannabis smokers and non-smokers

The prevalence of cannabis use was 18.2% (N = 34). Among cannabis smokers, 26.4% (n = 9) reported daily smoking, 32.3% (n = 11) weekly use, and 41.1% (n = 14) monthly use. Cannabis smokers had a rural background 79.4% with (p = 0.02), were either daily tobacco smokers 8.8% or ex-smokers 67.6% (p < 0.001). However, the difference was not significant for age, marital status, level of study, occupation, monthly income, etiology of ESKD, BMI, dialysis variables and treatment. The other measures are presented in Table 1. Cannabis smokers showed a higher prevalence of 25 OH Vitamin D deficiency compared to non-smokers (p = 0.009). Additionally, the serum concentration of HB was significantly elevated in cannabis smokers (p = 0.005). However, there were no significant differences in levels of CRP and PTH between cannabis smokers and non-smokers. See Table 2.

#### Cannabis and chronic pain

Cannabis smokers were less likely to have chronic pain, with a prevalence of 26.5% among smokers compared to 55.9% of non-smokers (p = 0.002). The pain intensity score and the prevalence of lower limb pain and shoulder pain were significantly lower in the cannabis smoker group (p < 0.05). Table 3 displays the chronic pain characteristics of cannabis smokers and non-smokers undergoing hemodialysis.

**Table 3:** Chronic pain characteristics of cannabis smokers and non-smokers undergoing hemodialysis (N = 186)

Pain characteristics	Non-smokers (n = 152)	Smokers (n = 34)	P value
Chronic pain			
No	67(44.1)	25 (73.5)	**0.002**
Yes	85(55.9)	9(26.5)	
Pain intensity score median (IQR)	4(6)	0(3)	0.006*
Painful sites			
Upper limbs	7(4.6)	0(0)	0.35
Lower limbs	49(32.2)	5(14.7)	**0.042**
Knee	31(91.2)	3(8.8)	1
Hip	28(82.4)	6(17.6)	0.71
Shoulders	34(100)	0(0)	**0.016**
Back	30(88.2)	4(11.8)	0.24
Chest	3(2)	0(0)	1
Number of painful sites median (IQR)	1(2)	0(1)	0.01*

Chi-square and Fisher tests

Significant result in bold, p < 0.05

^*^Mann–Whitney test

**Fig. 1:**
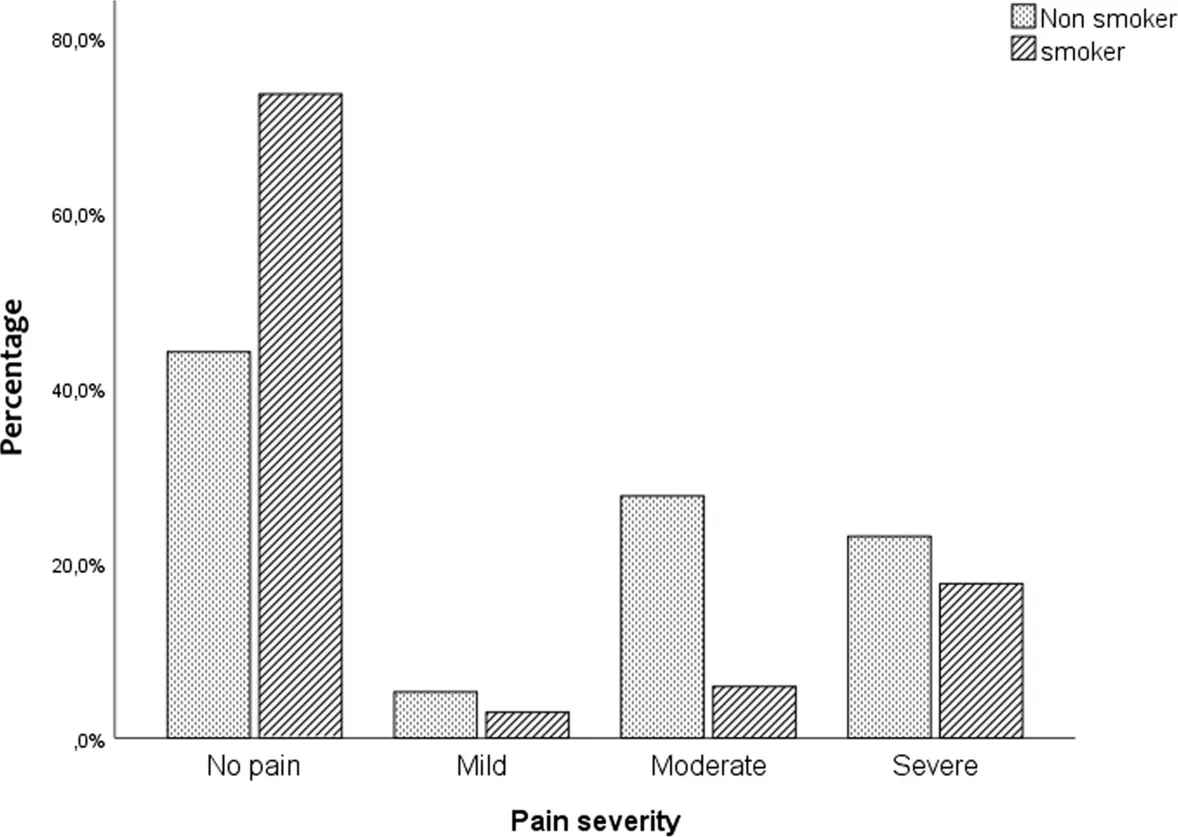
Pain severity of cannabis smokers and non-smokers undergoing hemodialysis (N = 186)

Among the 186 patients, 73.5% of cannabis smokers reported no chronic pain. Of the remaining 26.5%, 2.9% experienced mild pain, 5.9% had moderate pain and 17.6% suffered severe pain. However, in the non-smoking group, 44.1% reported no chronic pain, while 5.3%, 27.6% and 23% experienced mild, moderate and severe pain, respectively. Severe pain was more common among non-smokers than smokers’ group. Figure 1 shows the pain severity of cannabis smokers and non-smokers undergoing hemodialysis.

In the logistic regression analysis for controlling variables, the odd ratio of smoking cannabis on chronic pain in hemodialysis patients was OR = 0.202; 95% CI: [0.074. 0.549] . Table 4 displays chronic pain and cannabis smoking after controlling for confounding variables. .

**Table 4:** Chronic pain and cannabis smoking after controlling for confounding variables

	B	*P* value	Adjusted Odd Ratio	95% Confidence Interval	
				Lower Bound	Upper Bound
Cannabis smoker	−1,600	**0.002**	0.202	0.074	0.549
Constant	3.773	0.002	43.491		

Odd ratio adjusted for: Age, Residence, Vitamin D and Hemoglobin

#### Cannabis and quality of life

The mean overall score on the SF-36 was 47,06 ± 13,46. Concerning the Physical Component Summary (PCS), bodily pain recorded the highest score at 61.92 ± 27.18, while general health had the lowest score at 34.81 ± 18.04. For the Mental Component Summary (MCS), the highest score was for role emotional at 49.94 ± 18.07, whereas mental health recorded the lowest score at 38.42 ± 15.82. The Cronbach's Alpha for the SF-36 was found to be 0.794. The scores from the SF-36 are presented in Table 5.

**Table 5:** SF 36 subscales of patients undergoing hemodialysis (N = 186)

SF 36 subscales	N (mean ± SD)
Total score of 36 SF	47.06 ± 13.46
Physical Component Summary	
Physical functioning	56.10 ± 22.96
Role physical	53.09 ± 23.40
General Health	34.81 ± 18.04
Bodily pain	61.92 ± 27.18
Mental Component Summary	
Vitality	39.46 ± 22.81
Role emotional	49.94 ± 18.07
Mental health	38.42 ± 15.82
Social functioning	42.72 ± 17.47

*SF-36* Short-Form 36 questionnaire

In the sample, mean scores below 50 were observed in vitality, social functioning, mental health, role emotion and general health. The smokers' group had significantly higher mean scores in general health, bodily pain and vitality domains compared to the non-smokers' group (p < 0,05). Furthermore, the PCS score in the cannabis smokers’ group was higher than the non-smoker group (p = 0.003). Table 6 displays the quality of life of cannabis smokers and non smokers undergoing hemodialysis.

**Table 6:** SF 36 subscales of cannabis smokers and non-smokers undergoing hemodialysis (N = 186)

SF 36 subscales	Non-smokers (n = 152)	Smokers (n = 34)	*P* value
PCS	47.58 ± 23.58	60.77 ± 21.35	**0.003**
Physical functioning	55.30 ± 23.38	59.71 ± 20.96	0.031
Role physical	52.30 ± 23.43	56.62 ± 23.28	0.33
General Health	32.86 ± 17.81	43.53 ± 16.67	**0.02**
Bodily pain	58.84 ± 27.24	75.66 ± 22.58	** < 0.001**
MCS	49.13 ± 15.97	53.88 ± 17.93	0.12
Vitality	37.40 ± 22.64	48.68 ± 21.57	**0.009**
Role emotional	49.94 ± 17.97	49.95 ± 18.78	0.9
Mental health	38.54 ± 15.02	37.88 ± 19.22	0.85
Social functioning	42.58 ± 17.22	43.35 ± 18.78	0.81

*PCS* Physical Component Summary, *MCS* Mental Component Summary

Student t-test

Significant result in bold, *p* < 0.05

## Discussion

This study investigates the prevalence of recreational cannabis use among male hemodialysis patients and explores the differences in chronic pain characteristics and quality of life between cannabis users and non-users in the studied population. The findings reveal that a subset of male hemodialysis patients engage in recreational cannabis use. Notably, cannabis users were less likely to report chronic pain and more likely to experience a better quality of life compared to non-users. The analysis of these results and their implications are discussed in detail below.

The findings indicate that 18.2% of male hemodialysis patients reported cannabis use within the past three months. A similar study conducted in Canada revealed that 21% of hemodialysis patients used cannabis products, of which 91% use it for recreational purposes and 73% consume it illegally, with smoking being the most common method of consumption (64%) (Ho et al. 2024). Likewise, another study evaluating cannabis use among CKD patients and hemodialysis patients showed a significant prevalence of cannabis use in this population for recreational reasons, which was 52.5% (Collister et al. 2023). In this study, the prevalence of cannabis use was higher among individuals in rural communities. In contrast, Coughlin’s research found that cannabis use was more common in urban areas than in rural areas in the United States (Coughlin et al. 2019). The disparity in cannabis consumption between urban and rural areas in this present study may be related to the cultivation of cannabis in rural areas in northern Morocco (UNODC 2003), leading to its increased availability and accessibility in these locations. Besides, a study has shown that cannabis consumption and plantation in rural areas are linked to financial difficulties (Samarakoon et al. 2018).

In this study, the prevalence of tobacco smoking in the cannabis smokers’ group was 8.8%. The latter was less than the prevalence of current smoking in the general Moroccan population, which was 16% (Serhier et al. 2018).

When comparing laboratory parameters between cannabis smokers and non-smokers, a higher HB concentration and a lower 25 OH Vit D were observed in the smoker group. The findings of the studies have shown that cannabis consumption was significantly associated with elevated HB concentrations of 0.111 higher than never use (Zhang et al. 2009). In addition, research reported the relationship between 25 OH Vit D and cannabis use, revealing a lower level of 25 OH Vit in cannabis users with 5.20 nmol/l in difference versus less or non-users (Bemanian et al. 2022), which is consistent with this study.

Within this research, a relationship was established between cannabis smoking and chronic pain. Cannabis smokers were less likely to have chronic pain compared to non-smokers and the intensity of this pain was lower in cannabis users. These results are linked with several research. Cannabis was shown to be useful for medical purposes, proving its effectiveness in chronic nonmalignant neuropathic pain (Boychuk et al. 2015). In a meta-analysis study assessing the effects of cannabinoids on subjective pain, the results revealed that cannabinoids were associated with chronic pain (Yanes et al. 2019). In addition, an umbrella review of meta-analyses of randomised controlled trials and observational studies of risks and benefits of cannabis revealed that cannabis-based medicines could be considered for chronic pain across different conditions. For chronic pain, cannabinoids or the cannabis-based medicines reduced pain by 30% (high Grading of Recommendations Assessment, Development, and Evaluation) (Solmi et al. 2023), which could explain our results. The mechanism of action of cannabinoids can explain this relationship. Cannabinoid receptors CB1 and CB2 are present in spinal, supraspinal and peripheral sites, including analgesic processing (Richardson 2000). Their interaction with THC (endogenous and phytocannabinoids) can modulate basal and hyperalgesia thresholds of nociception (Richardson 2000; Narouze 2021). The mechanism of action likely involves presynaptic neurotransmitter and neuropeptide release inhibition, modification of postsynaptic neuronal excitability, activation of the descending pain inhibitory pathway and a decrease in neuroinflammatory signs (Starowicz and Finn 2017).

In terms of quality of life, the PCS was higher in cannabis smokers. In addition, the general health, bodily pain and vitality subscales were significantly greater in smokers than in the non-smokers group. In a study of recreational cannabis users in Brazil (Morais et al. 2022), the quality of life scores were higher for usual cannabis users compared to non-users, who reported more symptoms of depression or anxiety (Morais et al. 2022). Similarly, a study's results revealed a clinically significant improvement in overall quality of life in individuals with chronic conditions within three months of receiving medical cannabis (Tait et al. 2023). The outcomes of quality-of-life subscales differ from one study to another. A study examined the relationship between recreational cannabis use and quality of life and psychosocial results in head and neck cancer patients. The finding showed that pain/discomfort, as well as anxiety/depression, were improved among the cannabis user group (Zhang et al. 2018). Likewise, additional research, which examined the effects of medical cannabis on the quality of life of patients suffering from chronic illnesses, indicated an improvement in their energy levels, emotional well-being and physical and social functioning during the first 30 to 60 days of using medical cannabis (Kelley et al. 2024). Furthermore, patients using these drugs once a day were more likely to have better general health than twice or more times a day (Kelley et al. 2024).

In this study, the increased quality of life observed in the cannabis smoking group could potentially be attributed to the high HB levels and low intensity of chronic pain experienced. The study conducted by Al Jabi demonstrated a positive and significant association between HB levels and quality of life (Al-Jabi et al. 2023). In addition, other research indicated that hemodialysis patients with lower pain severity reported higher quality of life scores (Samoudi et al. 2021).

This finding represents the first step in a new approach to medical practice, in particular, the use of cannabis inhalation in the treatment of chronic pain in patients undergoing hemodialysis. Otherwise, the adverse effects of cannabis, particularly with cannabis smoking, could be detrimental to respiratory health, including lung function changes, lung destruction and significantly increasing risks of Chronic Obstructive Pulmonary Disease (Kaplan 2021). The recommendation of smoking cannabinoids in the treatment of chronic pain in hemodialysis patients must be done with caution. In the context of the lack of efficacy of acetaminophen, non-steroidal anti-inflammatory drugs and opioids. Studies that investigate the relationship between recreational or medical cannabis use and chronic pain in patients undergoing hemodialysis, as well as the undesirable effects in this population, already exist (Collister et al. 2023; Kliuk-Ben Bassat et al. 2023). However, further studies should be carried out to investigate the relationship of frequency, quantity and quality of cannabis on efficiency and reduction of chronic pain in patients treated by hemodialysis.

The current research has several limitations. Firstly, the descriptive analysis study approach restricts the opportunity to derive the relationship and the effect size between cannabis and chronic pain. Secondly, the exclusively male composition of the sample and the use of a small sample from the same geographic region constitute limitations of this study, thereby restricting the generalizability of the findings to the overall hemodialysis population. Thirdly, legal problems and the suffering from pain reduce the opportunity for consumption and the possibility of access to cannabis. Lastly, data on cannabis CBD/THC potency was not taken into account. Nevertheless, it is the first study in Africa that describes the users of psychoactive drugs in hemodialysis patients and investigates pain and quality of life related to cannabis use.

## Conclusions

Cannabis smoking was the lifestyle of some males in hemodialysis patients. Comparing sociodemographic, clinical, dialysis and biological variables, cannabis smokers were predominantly from rural areas, had a history of tobacco use and were less likely to suffer from chronic pain than non-smokers. The pain was less severe in the smokers' group and the number of painful sites was lower. Furthermore, this group had a better quality of life. However, more research is needed to investigate the relationship of frequency, quantity and quality of cannabis on efficiency and reduction of chronic pain in patients treated by hemodialysis.

## Data Availability

The datasets used and analyzed during the current study are available from the corresponding author on reasonable request.

## Abbreviations

THC: Δ-9-Tetrahydrocannabinol
CBD: Cannabidiol
ESKD: End Stage Kidney Disease
AVF: Arterial Venous Fistula
BMI: Body Mass Index
25 OH Vit D: 25-hydroxyvitamin D
HB: Hemoglobin
CRP: C-Reactive Protein
PTH: Parathyroid Hormone
SF-36: Short-Form 36 questionnaire
PCS: Physical Component Summary
MCS: Mental Component Summary
